# Association of cumulative non-high-density lipoprotein cholesterol to high-density lipoprotein cholesterol ratio with the risk of cardiometabolic disease

**DOI:** 10.3389/fcvm.2024.1500025

**Published:** 2024-11-20

**Authors:** Luqing Liu, Shihe Liu, Yicheng Liao, Xiaoxue Zhang, Meixiao Wang, Liming Lin, Chenrui Zhu, Shouling Wu, Yuntao Wu

**Affiliations:** ^1^ Department of Cardiology, Kailuan General Hospital Affiliated to North China University of Science and Technology, Tangshan, China; ^2^ Graduate School, North China University of Science and Technology, Tangshan, China

**Keywords:** cardiometabolic disease, cumulative exposure, non-high-density lipoprotein cholesterol to high-density lipoprotein cholesterol ratio, epidemiology, influencing factors

## Abstract

**Background:**

One measurement of non-high-density lipoprotein cholesterol to high-density lipoprotein cholesterol ratio (NHHR) is considered to be associated with insulin resistance and cardiovascular disease (CVD) risk. This study aimed to exploring the association between cumulative non-high-density lipoprotein cholesterol/high-density lipoprotein cholesterol (Cum NHHR) exposure levels and the risk of cardiometabolic disease (CMD).

**Methods:**

This prospective cohort study included 43,735 participants, who participated in three consecutive health examinations in 2006, 2008, 2010 and had no history of CMD or cancer. The participants were divided into quartiles bases on their cum NHHR. Multivariate Cox proportional hazards model was used to assess the association between cum NHHR and the risk of CMD. Additionally, the direct method of standardized ratios was employed to calculate the absolute risk of CMD attributable to cum NHHR.

**Results:**

Over a median follow-up period of 10.92 years (IQR: 10.22–11.26 years), 7,388 participants were newly diagnosed with CMD. In the multivariate-adjusted model, participants in quartiles Q2, Q3 and Q4 showed a progressively increased relative risk of CMD compared to those in Q1, The fully adjusted hazard ratios (95% confidence intervals) for the risk of CMD in the Q2, Q3, and Q4 groups were 1.11 (1.04–1.20), 1.23 (1.14–1.32), and 1.29 (1.20–1.38), respectively, compared with the Q1 group. This association remained significant even after further adjustment for single measurements of NHHR. Moreover, cum NHHR was positively correlated with the absolute risk of CMD, cardiovascular diseases (CVD), and type 2 diabetes (T2DM).

**Conclusions:**

Higher cum NHHR is significantly associated with an increased risk of CMD, independent of single-point NHHR level. Additionally, there are significant different strengths of correlations between cum NHHR and different diseases.

## Introduction

Cardiometabolic disease (CMD), characterized by metabolic disorders with insulin resistance at their core, include cardiovascular diseases and type 2 diabetes (1). Since 1990, the global prevalence of CMD has risen from 456 million to 989 million by 2019 (2–4). According to the 2021 Global Burden of Disease report, coronary heart disease, stroke, and type 2 diabetes ranked as the first, third, and tenth leading causes of age-standardized mortality worldwide, respectively—all major forms of CMD (5). As CMD has become a significant global public health issue, early identification of high-risk populations and control of modifiable risk factors are crucial for the prevention and management of CMD.

Dyslipidemia is a well-established, modifiable risk factor for CMD (6). Compared with traditional lipid markers such as low-density lipoprotein cholesterol (LDL-C) and high-density lipoprotein cholesterol (HDL-C), the non-high-density lipoprotein cholesterol/high-density lipoprotein cholesterol ratio (NHHR) has been shown to be a better predictor of metabolic risk and insulin resistance. It is more strongly associated with the risk of cardiovascular diseases, cerebrovascular diseases (7–9), and diabetes (10, 11). For instance, Kouvari et al. found that for every 1-unit increase in NHHR, the risk of cardiovascular diseases increased by approximately 15% (12). Similarly, Chen et al. discovered that each 1-standard deviation increase in log_10_ NHHR was associated with a 10% increase in diabetes risk (10). However, previous studies had smaller sample sizes and focused primarily on either cardiovascular diseases or diabetes, with no large cohort studies exploring the relationship between NHHR levels and CMD risk as a whole. Moreover, single-point measurements of NHHR fail to capture long-term exposure levels. Thus, this study, based on the Kailuan cohort (Registration No: ChiCTR-TNC-11001489), aims to investigate the impact of cumulative non-HDL-C/HDL-C (cum NHHR) exposure on CMD incidence, providing crucial evidence for the development of new prevention and treatment strategies for CMD.

## Subjects and methods

### Research subjects

The Kailuan Study is a large-scale, prospective cohort study based on a functional community population. From 2006 to 2007, the Kailuan General Hospital and its 10 affiliated hospitals conducted the first round of health examinations on active employees and retired personnel of the Kailuan Group, during which relevant data were collected. Since then, follow-up health examinations have been conducted every two years, as described in previous publications from this research group (13). Since 2006, annual follow-up has been conducted for cardiovascular and cerebrovascular events as well as all-cause mortality. This study is based on the Kailuan Study and selected participants who underwent three consecutive health examinations from 2006 to 2010 as the observation cohort. The inclusion criteria were: (1) individuals who participated in three consecutive health examinations in the Kailuan Study cohort between 2006 and 2010; and (2) individuals who provided informed consent and agreed to participate in the Kailuan Study. The exclusion criteria at the time of the 2010 health examination were: (1) participants with a history of cardiometabolic diseases; (2) participants with a history of cancer; and (3) participants with missing total cholesterol or high-density lipoprotein cholesterol data in any of the three health examinations. This study was approved by the Ethics Committee of Kailuan General Hospital. [(2006) Ethics Approval No. 5].

### Data collection

Demographic data of the participants (e.g., birthdate, gender), personal lifestyle habits (e.g., smoking, alcohol intake), anthropometric measurements (e.g., height, weight), personal medical history (e.g., hypertension, diabetes), medication use (e.g., antihypertensive drugs, lipid-lowering drugs), blood pressure, and biochemical test results were collected. A detailed description of these variables can be found in previous publications by this research group (13). Biochemical measurements were taken after fasting for more than 8 h, with venous blood collected and centrifuged to obtain serum for the detection of total cholesterol (TC), high-density lipoprotein cholesterol (HDL-C), low-density lipoprotein cholesterol (LDL-C), triglycerides (TG), fasting blood glucose (FBG), and hypersensitive C-reactive protein (hs-CRP). The NHHR was calculated as the ratio of the sum of all cholesterol components except HDL-C to HDL-C itself (14), using the formula: NHHR = (TC - HDL-C)/HDL-C. Biochemical indicators were measured using a Hitachi 7,600 automatic biochemical analyzer. Estimated glomerular filtration rate (eGFR) was calculated using the formula from the Chronic Kidney Disease Epidemiology Collaboration (CKD-EPI) equation (15). All procedures were performed in strict accordance with the reagent manufacturer's instructions and were conducted by professional laboratory technicians.

### Relevant definitions and grouping

cum NHHR represents long-term cumulative exposure to NHHR and is calculated as follows (16): cum NHHR = [(NHHR_1_ + NHHR_2_)/2 × time_1–2_] + [(NHHR_2_ + NHHR_3_)/2 × time_2–3_].

Where NHHR_1_, NHHR_2_, and NHHR_3_ refer to the values of NHHR at the first (2006), second (2008), and third (2010) health examinations, respectively. Time_1–2_ and time_2–3_ refer to the intervals between the first and second, and the second and third health examinations, respectively.

Hypertension was defined as systolic blood pressure (SBP) ≥140 mmHg (1 mmHg = 0.133 kPa) and/or diastolic blood pressure (DBP) ≥90 mmHg; or the use of antihypertensive medications or a history of hypertension, even if SBP <140 mmHg and DBP <90 mmHg. Diabetes was defined as fasting blood glucose ≥7.0 mmol/L, or the use of antidiabetic medications or a history of diabetes, even if fasting blood glucose <7.0 mmol/L. Smoking was defined as a history of smoking or current smoking (at least one cigarette per day on average for ≥1 year). Alcohol consumption was defined as a history of drinking or current drinking [at least 100 mL of liquor (alcohol content >50%) per day on average for ≥1 year]. Physical exercise was defined as exercising ≥3 times per week, with each session lasting ≥30 min.

### Assessment of outcomes

CMD include cardiovascular diseases (CVD) and type 2 diabetes (T2DM) (17). With CVD encompassing myocardial infarction, revascularization, ischemic stroke, cerebral hemorrhage, and subarachnoid hemorrhage. ICD codes from the Tenth Revisions (ICD-10) were used to identify diagnoses of CMD (Supplementary Table S1). All of the aforementioned events were confirmed by professional physicians through hospitalization records.

The follow-up period began at the date of the 2010 health examination and continued until the occurrence of CMD or all-cause mortality, with the follow-up endpoint set at December 31, 2021, for participants without events. For individuals experiencing multiple CMD events, the first event and its corresponding time were considered as the primary outcome. In the specific analysis of CMD endpoints, if a participant experienced two or more events, each event was recorded with its respective time and outcome. Data on all-cause mortality were collected through the social security system.

### Statistical analysis

Data analyses were performed using SAS 9.4 statistical software (SAS Institute, Cary, North Carolina). Normally distributed continuous variables were expressed as mean ± standard deviation (x̅ ± S) and compared between groups using one-way analysis of variance (ANOVA). Non-normally distributed continuous variables were expressed as median (*P*25, *P*75), and group comparisons were performed using the Kruskal-Wallis non-parametric test. Categorical variables were expressed as relative frequencies, and comparisons between groups were made using the χ^2^ test.

Multivariate Cox proportional hazards regression models were used to assess the impact of cum NHHR quartiles and each standard deviation increase on CMD risk. Restricted cubic spline models were employed to evaluate potential non-linear relationships between cum NHHR and CMD risk. Kaplan-Meier curves were used to calculate cumulative incidence rates of CMD, and group comparisons were made using the Log-rank test. The direct method of standardized ratios was applied to calculate the absolute risk (AR) of CMD, CVD, and diabetes associated with cum NHHR exposure.

For specific CMD endpoint analyses, competing risk models were used to explore the association between cum NHHR quartiles and each standard deviation increase with the risk of myocardial infarction, revascularization, ischemic stroke, cerebral hemorrhage, subarachnoid hemorrhage, and type 2 diabetes, with death treated as a competing event. In sensitivity analyses, competing risk models were applied to assess the impact of cum NHHR quartiles and each standard deviation increase on CMD risk. Additional sensitivity analyses excluded individuals with follow-up periods of less than one year, those using lipid-lowering medications, those with a history of atrial fibrillation, and those with a history of heart failure, to validate the association between cum NHHR quartiles and CMD. A two-sided *P*-value of <0.05 was considered statistically significant.

## Results

### Characteristics of the study participants

A total of 58,869 participants underwent three consecutive health examinations between 2006 and 2010 as part of the Kailuan Study. After excluding 11,811 individuals with a history of CMD at the 2010 health examination, 532 individuals with a history of cancer, and 2,791 individuals with missing data on total cholesterol (TC) or high-density lipoprotein cholesterol (HDL-C), 43,735 participants were ultimately included in the analysis. The mean age of the participants was 52.05 ± 12.10 years, and 32,849 (75.11%) were male.

cum NHHR values were divided into quartiles: Q1 (cum NHHR < 7.55), Q2 (7.55 ≤ cum NHHR < 9.27), Q3 (9.27 ≤ cum NHHR < 11.24), and Q4 (cum NHHR ≥ 11.24). As cum NHHR exposure increased, significant increases were observed in mean age, systolic blood pressure, diastolic blood pressure, body mass index (BMI), fasting blood glucose, triglycerides, total cholesterol, low-density lipoprotein cholesterol (LDL-C), and hypersensitive C-reactive protein (hs-CRP) levels. Furthermore, the proportion of males, participants engaging in physical exercise, individuals with hypertension, and those using antihypertensive and lipid-lowering medications were significantly higher in the group with high cum NHHR exposure, with all differences being statistically significant (all *P* < 0.05, Table 1).

**Table 1:** General characteristics of the participants.

	Total	Q1	Q2	Q3	Q4	*P*
Participants	43,735	10,933	10,934	10,934	10,934	
Age, year	52.05 ± 12.10	49.97 ± 11.81	50.59 ± 11.90	52.26 ± 12.13	55.37 ± 11.82	<0.01
Male, *N* (%)	32,849 (75.11)	7,429 (67.95)	8,258 (75.53)	8,551 (78.21)	8,611 (78.75)	<0.01
SBP, mmHg	129.24 ± 18.18	125.74 ± 17.56	128.20 ± 17.56	130.04 ± 17.80	132.98 ± 19.01	<0.01
DBP, mmHg	83.96 ± 10.20	82.18 ± 9.93	83.73 ± 10.09	84.48 ± 10.00	85.44 ± 10.47	<0.01
BMI, kg/m^2^	24.91 ± 3.29	23.73 ± 3.15	24.68 ± 3.23	25.33 ± 3.20	25.92 ± 3.16	<0.01
FBG, mmol/L	5.24 ± 0.59	5.12 ± 0.58	5.22 ± 0.58	5.28 ± 0.59	5.33 ± 0.60	<0.01
TG, mmol/L	1.25 (0.89–1.83)	0.95 (0.69–1.34)	1.19 (0.88–1.65)	1.34 (0.99–1.93)	1.64 (1.16–2.42)	<0.01
TC, mmol/L	4.96 ± 0.94	4.58 ± 0.84	4.82 ± 0.84	5.02 ± 0.89	5.42 ± 0.97	<0.01
HDL-C, mmol/L (mmol/L)	1.57 ± 0.44	1.85 ± 0.46	1.60 ± 0.40	1.48 ± 0.37	1.35 ± 0.34	<0.01
LDL-C, mmol/L (mmol/L)	2.58 ± 0.77	2.22 ± 0.68	2.53 ± 0.65	2.68 ± 0.72	2.87 ± 0.85	<0.01
NHHR_1_	2.31 ± 0.87	1.74 ± 0.56	2.08 ± 0.67	2.41 ± 0.74	3.00 ± 0.91	<0.01
NHHR_2_	2.42 ± 0.86	1.58 ± 0.42	2.20 ± 0.44	2.59 ± 0.48	3.33 ± 0.85	<0.01
NHHR_3_	2.35 ± 0.96	1.57 ± 0.58	2.12 ± 0.66	2.53 ± 0.75	3.19 ± 0.96	<0.01
eGFR, mL/(min·1.73^2^)] [mL/(min·1.73 m^2^)]	91.35 ± 18.40	95.33 ± 18.20	91.17 ± 19.18	90.48 ± 18.30	88.41 ± 17.17	<0.01
hs-CRP, mg/L	1.00 (0.50–2.40)	0.95 (0.50–2.20)	0.92 (0.40–2.25)	1.00 (0.43–2.30)	1.30 (0.65–2.80)	<0.01
smoking, *N* (%)	16,704 (38.19)	4,079 (37.31)	4,064 (37.17)	4,230 (38.69)	4,331 (39.61)	<0.01
alcohol intake, *N* (%)	15,600 (35.67)	4,007 (36.65)	3,832 (35.05)	3,963 (36.24)	3,798 (34.74)	0.07
Physical exercisers, *N* (%)	6,122 (14.00)	1,438 (13.15)	1,361 (12.45)	1,500 (13.72)	1,823 (16.67)	<0.01
Hypertension, *N* (%)	18,134 (41.46)	3,686 (33.71)	4,210 (38.50)	4,754 (43.48)	5,484 (50.16)	<0.01
Antihypertensive drugs, *N* (%)	4,224 (9.66)	749 (6.85)	784 (7.17)	1,052 (9.62)	1,639 (14.99)	<0.01
Lipid-lowering drugs, *N* (%)	281 (0.64)	33 (0.30)	49 (0.45)	91 (0.83)	108 (0.99)	<0.01

*P*, comparison of General Characteristics between different Cum NHHR Quartiles.

SBP, systolic blood pressure; DBP, diastolic blood pressure; BMI, body mass index; FBG, fasting blood glucose; TC total cholesterol; LDL-C, low-density lipoprotein cholesterol; HDL-C, high-density lipoprotein cholesterol; hs-CRP, high-sensitivity C reactive protein; TG, triglyceride; eGFR, estimated glomerular filtration rate.

NHHR_1_, NHHR_2_, and NHHR_3_ refer to the NHHR values from the first (2006/2007), second (2008/2009), and third (2010/2011) health examinations of the study participants, respectively.

### Incidence density and cumulative incidence rate

**Figure 1:**
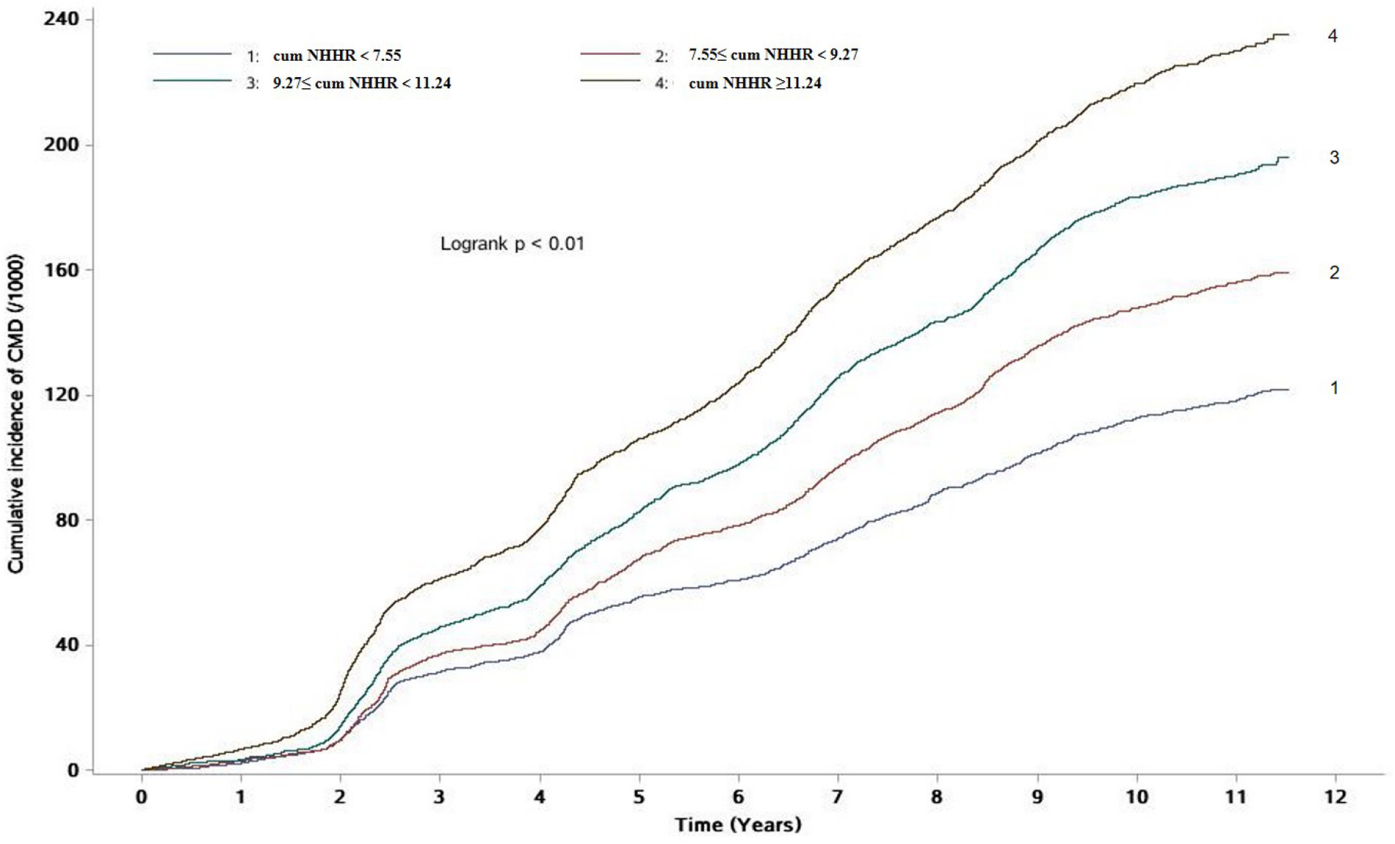
Cumulative incidence of CMD in different Cum NHHR quartile groups.

During a median follow-up of 10.92 (IQR: 10.22–11.26) years, 7,388 new cases of CMD were identified. The incidence densities of CMD in the Q1–Q4 groups were 11.41, 15.25, 19.11, and 23.84 per 1,000 person-years, respectively. The cumulative incidence rates of CMD in these groups were 11.81%, 15.60%, 19.04%, and 22.99%, respectively. The Log-rank test indicated that the cumulative incidence rates between the four groups were statistically significant (χ^2^ = 513.65, *P* < 0.01, Table 2 and Figure 1).

**Table 2:** Cox regression analysis of the impact of different Cum NHHR quartiles on CMD.

	Cases, *N* (%)	Incidence density/10^3^ person-years	HR (95% CI)			
			Model 1	Model 2	Model 3	Model 4
CMD						
Per SD			1.22 (1.20–1.25)	1.10 (1.07–1.12)	1.10 (1.07–1.13)	1.10 (1.08–1.13)
Q1	1,274 (11.65)	11.41	1.00	1.00	1.00	1.00
Q2	1,668 (15.26)	15.25	1.29 (1.20–1.38)	1.11 (1.04–1.20)	1.11 (1.04–1.20)	1.11 (1.03–1.20)
Q3	2,026 (18.53)	19.11	1.54 (1.44–1.66)	1.23 (1.15–1.33)	1.23 (1.14–1.32)	1.22 (1.14–1.32)
Q4	2,420 (22.13)	23.84	1.79 (1.67–1.92)	1.30 (1.21–1.40)	1.29 (1.20–1.38)	1.28 (1.18–1.39)
*P* for trend			<0.01	<0.01	<0.01	<0.01

Model 1: adjusted for age (continuous) and gender (male or female).

Model2: included variables in model 1 and further smoking (yes or no), alcohol intake (yes or no), Physical exercisers (yes or no), BMI (continuous), SBP (continuous), FBG (continuous), eGFR (continuous), hs-CRP (continuous).

Model 3: included variables in model 2 and further Antihypertensive drugs (yes or no), Lipid-lowering drugs (yes or no).

Model 4: included variables in model 3 and further NHHR_1_ (the NHHR value from the 2006/2007 health examination).

### Association between cum NHHR and the risk of CMD

Using the occurrence of CMD as the dependent variable, the following models were constructed: Model 1 adjusted for age and gender; Model 2 further adjusted for smoking, alcohol intake, physical exercise, body mass index, systolic blood pressure, fasting blood glucose, glomerular filtration rate, and hypersensitive C-reactive protein based on Model 1; Model 3 additionally adjusted for the use of antihypertensive drugs and Lipid-lowering drugs based on Model 2; and Model 4 further adjusted for NHHR values at the 2006 health examination based on Model 3.

**Figure 2:**
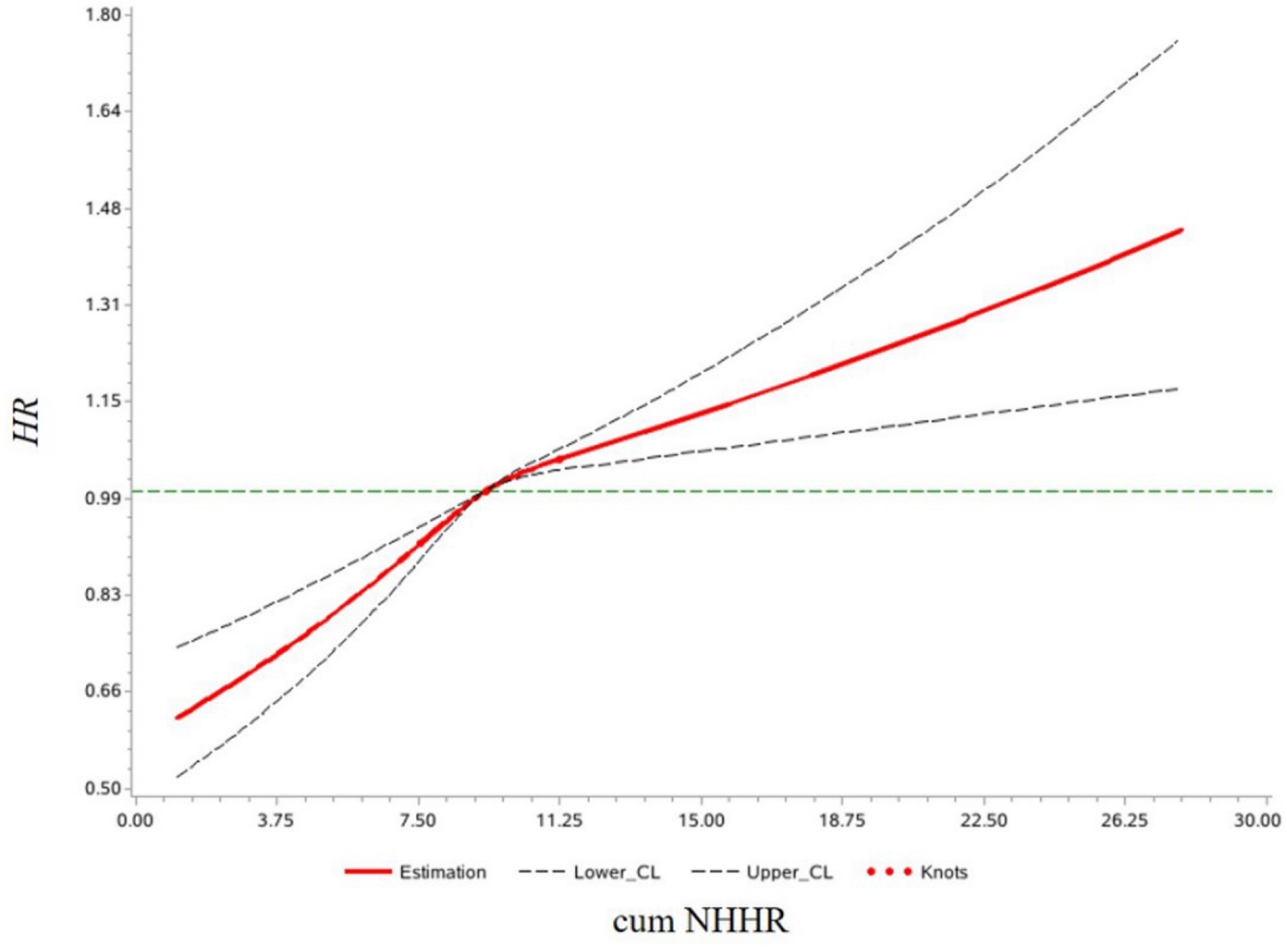
Restricted cubic spline plot of Cum NHHR and CMD risk.

Multivariate Cox proportional hazards regression analysis showed that, in Model 3, compared to the Q1 group, the risk of developing CMD increased by 11% (HR: 1.11, 95% CI: 1.04–1.20), 23% (HR: 1.23, 95% CI: 1.14–1.32), and 29% (HR: 1.29, 95% CI: 1.20–1.38) in the Q2, Q3, and Q4 groups, respectively. For each 1-standard deviation increase in cum NHHR, the risk of CMD increased by 10% (HR: 1.10, 95% CI: 1.07–1.13). In Model 4, after adjusting for NHHR values from the 2006/2007 health examination, the results remained consistent with Model 3, as shown in Table 2. Analysis results of competing risk models showed that, In Model 4, For each 1-standard deviation increase in cum NHHR, the risk of CMD increased by 10% (HR: 1.10, 95% CI: 1.07–1.14), which further demonstrated the robustness of the results of this study, as shown in Table 3. Restricted cubic spline analysis demonstrated a significant relationship between cum NHHR exposure and CMD risk (overall association *P* < 0.0001, non-linear association *P* = 0.0055, Figure 2).

**Table 3:** Competing risk model.

	Model 1 HR (95% CI)	Model 2 HR (95% CI)	Model 3 HR (95% CI)	Model 4 HR (95% CI)
CMD				
Per SD	1.23 (1.20–1.25)	1.11 (1.08–1.13)	1.10 (1.07–1.13)	1.10 (1.07–1.14)
Q1	1.00	1.00	1.00	1.00
Q2	1.29 (1.20–1.39)	1.12 (1.04–1.21)	1.12 (1.04–1.21)	1.12 (1.04–1.21)
Q3	1.55 (1.45–1.67)	1.24 (1.15–1.33)	1.23 (1.15–1.33)	1.23 (1.14–1.33)
Q4	1.81 (1.69–1.94)	1.32 (1.23–1.41)	1.30 (1.21–1.40)	1.29 (1.19–1.40)
*P* for trend	<0.01	<0.01	<0.01	<0.01

Model 1: adjusted for age (continuous) and gender (male or female).

Model 2: included variables in model 1 and further smoking (yes or no), alcohol intake (yes or no), Physical exercisers (yes or no), BMI (continuous), SBP (continuous), FBG (continuous), eGFR (continuous), hs-CRP (continuous).

Model 3: included variables in model 2 and further Antihypertensive drugs (yes or no), Lipid-lowering drugs (yes or no).

Model 4: included variables in model 3 and further NHHR_1_ (the NHHR value from the 2006/2007 health examination).

The 5-year absolute risk of CMD for the Q1 to Q4 groups was 5.84%, 6.86%, 8.08%, and 9.80%, respectively, while the 5-year absolute risk of CVD was 1.98%, 2.47%, 2.88%, and 3.97%, and the 5-year absolute risk of T2DM was 4.39%, 5.12%, 5.89%, and 7.01%. The 10-year absolute risk of CMD for the Q1 to Q4 groups was 11.70%, 14.68%, 17.58%, and 20.40%, respectively, while the 10-year absolute risk of CVD was 4.90%, 5.99%, 7.06%, and 8.62%, and the 10-year absolute risk of T2DM was 7.47%, 9.67%, 11.55%, and 13.42% (Table 4).

**Table 4:** Absolute risk of endpoint events across different cumulative NHHR quartiles.

	AR (95% CI)		
	CMD	CVD	T2DM
5-year			
Q1	5.84% (5.37%–6.31%)	1.98% (1.71%–2.26%)	4.39% (3.98%–4.79%)
Q2	6.86% (6.36%–7.36%)	2.47% (2.17%–2.77%)	5.12% (4.69%–5.55%)
Q3	8.08% (7.55%–8.61%)	2.88% (2.56%–3.19%)	5.89% (5.43%–6.34%)
Q4	9.80% (9.23%–10.38%)	3.97% (3.62%–4.33%)	7.01% (6.51%–7.50%)
*P*-value	<0.01	<0.01	<0.01
10-year			
Q1	11.70% (11.04%–12.37%)	4.90% (4.47%–5.34%)	7.47% (6.95%–8.00%)
Q2	14.68% (13.96%–15.41%)	5.99% (5.53%–6.46%)	9.67% (9.08%–10.26%)
Q3	17.58% (16.80%–18.37%)	7.06% (6.57%–7.56%)	11.55% (10.92%–12.18%)
Q4	20.40% (19.55%–21.25%)	8.62% (8.09%–9.15%)	13.42% (12.72%–14.12%)
*P*-value	<0.01	<0.01	<0.01

Model adjusted for age (≥45years or <45years), gender (male or female).

### Stratified analysis

The results suggested a significant multiplicative interaction between cum NHHR exposure and gender (male or female), age (≥45 years or <45 years), and hypertension (yes or no) (*P* < 0.05). Compared to the Q1 group, the risk of developing CMD in the Q4 group increased by 31% (HR: 1.31, 95% CI: 1.12–1.53) for women, 35% (HR: 1.35, 95% CI: 1.13–1.63) for individuals under 45 years old, and 38% (HR: 1.38, 95% CI: 1.24–1.54) for those without hypertension. Meanwhile, in the Q4 group, the risk of CMD increased by 27% (HR: 1.27, 95% CI: 1.17–1.37) for men, 29% (HR: 1.29, 95% CI: 1.19–1.39) for individuals aged 45 years or older, and 21% (HR: 1.21, 95% CI: 1.10–1.33) for those with hypertension (Table 5).

**Table 5:** Stratified analysis.

	Cases, *N* (%)	HR (95% CI)					*P* for interaction
		Per SD	Q1	Q2	Q3	Q4	
Gender							<0.01
Male	6,057 (18.44)	1.09 (1.06–1.11)	1.00	1.13 (1.04–1.23)	1.25 (1.16–1.36)	1.27 (1.17–1.37)	
Female	1,331 (12.23)	1.13 (1.07–1.19)	1.00	1.00 (0.85–1.19)	1.05 (0.89–1.24)	1.31 (1.12–1.53)	
Age							<0.01
≥45 years	6,214 (19.39)	1.10 (1.07–1.12)	1.00	1.10 (1.01–1.19)	1.24 (1.15–1.34)	1.29 (1.19–1.39)	
<45 years	1,174 (10.04)	1.13 (1.06–1.21)	1.00	1.11 (0.93–1.32)	1.13 (0.95–1.35)	1.35 (1.13–1.63)	
Hypertension							<0.01
Yes	4,297 (23.70)	1.08 (1.05–1.11)	1.00	1.06 (0.96–1.17)	1.19 (1.08–1.30)	1.21 (1.10–1.33)	
No	3,091 (12.07)	1.13 (1.09–1.17)	1.00	1.16 (1.04–1.30)	1.24 (1.11–1.38)	1.38 (1.24–1.54)	
Smoking							0.66
Yes	3,135 (18.77)	1.08 (1.04–1.12)	1.00	1.08 (0.97–1.21)	1.24 (1.11–1.38)	1.26 (1.13–1.41)	
No	4,253 (15.73)	1.11 (1.08–1.14)	1.00	1.14 (1.04–1.26)	1.22 (1.11–1.35)	1.31 (1.20–1.44)	
Alcohol intake							0.71
Yes	2,754 (17.65)	1.07 (1.03–1.11)	1.00	1.12 (1.00–1.26)	1.20 (1.07–1.35)	1.24 (1.10–1.38)	
No	4,634 (16.47)	1.11 (1.08–1.14)	1.00	1.11 (1.01–1.22)	1.25 (1.14–1.37)	1.32 (1.21–1.45)	

Model adjusted for age (continuous), gender (male or female), smoking (yes or no), alcohol intake (yes or no), Physical exercisers (yes or no), BMI (continuous), SBP (continuous), FBG (continuous), eGFR (continuous), hs-CRP (continuous), Antihypertensive drugs (yes or no), Lipid-lowering drugs (yes or no).

### The impact of cum NHHR on different subtypes of CMD

**Figure 3:**
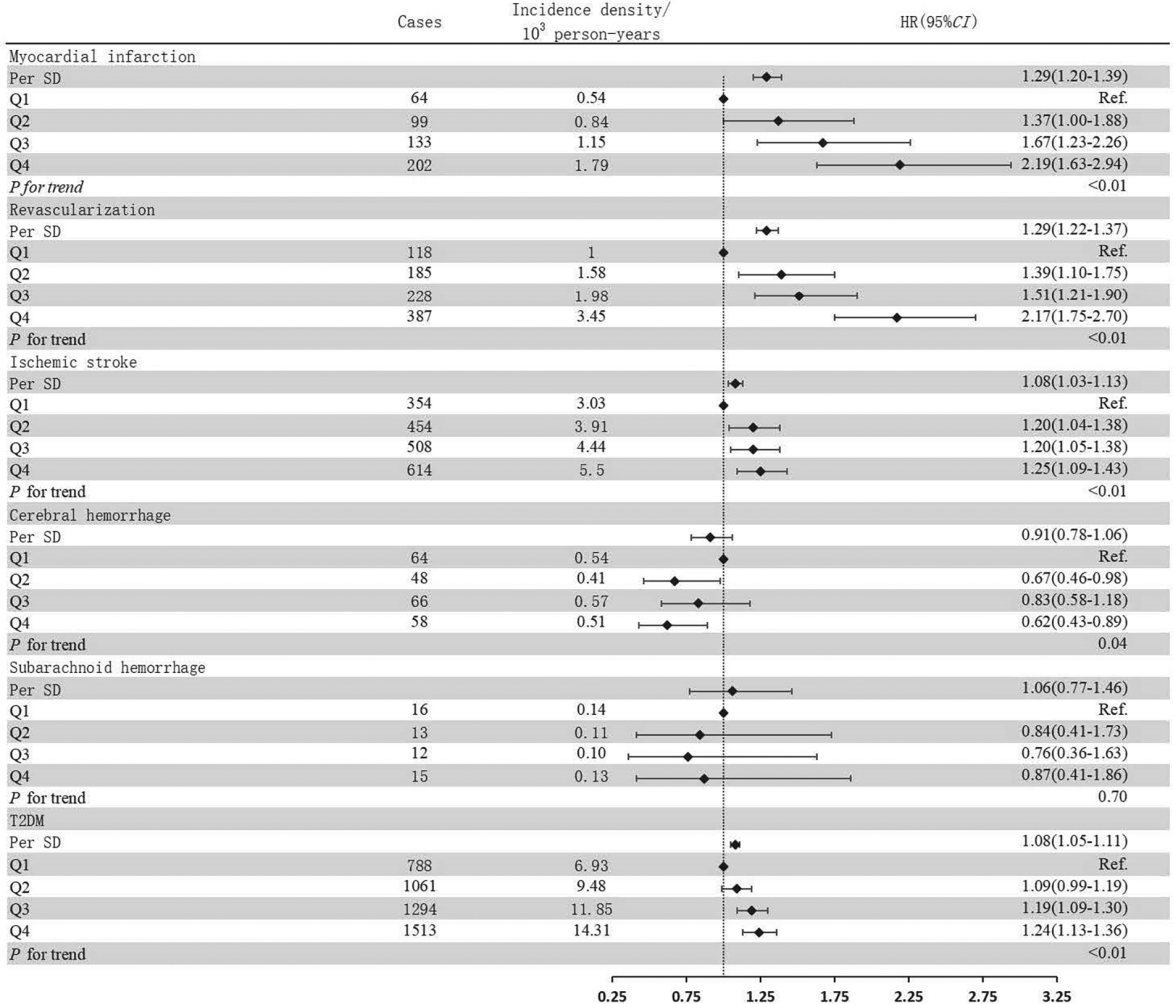
Impact of different Cum NHHR quartiles on CMD subtypes. Model adjusted for age (continuous), gender (male or female), smoking (yes or no), alcohol intake (yes or no), Physical exercisers (yes or no), BMI (continuous), SBP (continuous), FBG (continuous), eGFR (continuous), hs-CRP (continuous), Antihypertensive drugs (yes or no), Lipid-lowering drugs (yes or no).

Compared to the Q1 group, the risk of myocardial infarction increased by 37% (HR: 1.37, 95% CI: 1.00–1.88), 67% (HR: 1.67, 95% CI: 1.23–2.26), and 119% (HR: 2.19, 95% CI: 1.63–2.94) in the Q2, Q3, and Q4 groups, respectively. The risk of revascularization increased by 39% (HR: 1.39, 95% CI: 1.10–1.75), 51% (HR: 1.51, 95% CI: 1.21–1.90), and 117% (HR: 2.17, 95% CI: 1.75–2.70) in the Q2, Q3, and Q4 groups, respectively. The risk of ischemic stroke increased by 20% (HR: 1.20, 95% CI: 1.04–1.38), 20% (HR: 1.20, 95% CI: 1.05–1.38), and 25% (HR: 1.25, 95% CI: 1.09–1.43) in the Q2, Q3, and Q4 groups, respectively. There was no statistically significant difference in the risk of diabetes between the Q1 and Q2 groups, while the risk of diabetes increased by 19% (HR: 1.19, 95% CI: 1.09–1.30) and 24% (HR: 1.24, 95% CI: 1.13–1.36) in the Q3 and Q4 groups, respectively. cum NHHR exposure showed an approximate U-shaped relationship with the risk of cerebral hemorrhage. Compared to the Q1 group, there was no statistically significant difference in the Q3 group, while the risk of cerebral hemorrhage decreased by 33% (HR: 0.67, 95% CI: 0.46–0.98) and 38% (HR: 0.62, 95% CI: 0.43–0.89) in the Q2 and Q4 groups, respectively (Figure 3).

### Sensitivity analysis

After excluding participants with a follow-up period of less than one year, those taking lipid-lowering medications, and those with a history of atrial fibrillation or heart failure, we repeated the multivariable Cox regression analysis. The sensitivity analysis results were consistent with the main findings (Table 6).

**Table 6:** Sensitivity analysis.

	HR (95% CI)				
	Per SD	Q1	Q2	Q3	Q4
CMD					
Exclude those with a follow-up time of less than one year	1.09 (1.07–1.12)	1.00	1.11 (1.03–1.20)	1.23 (1.14–1.32)	1.28 (1.19–1.37)
Exclude individuals taking lipid-lowering drugs	1.09 (1.07–1.12)	1.00	1.11 (1.03–1.20)	1.23 (1.14–1.32)	1.28 (1.19–1.37)
Exclude individuals with a history of atrial fibrillation	1.10 (1.07–1.12)	1.00	1.11 (1.03–1.20)	1.23 (1.14–1.32)	1.29 (1.20–1.38)
Exclude individuals with a history of heart failure	1.09 (1.07–1.12)	1.00	1.10 (1.02–1.18)	1.22 (1.13–1.31)	1.26 (1.18–1.36)

Model adjusted for age (continuous), gender (male or female), smoking (yes or no), alcohol intake (yes or no), Physical exercisers (yes or no), BMI (continuous), SBP (continuous), FBG (continuous), eGFR (continuous), hs-CRP (continuous), Antihypertensive drugs (yes or no), Lipid-lowering drugs (yes or no).

## Discussion

The primary finding of this study is that cum NHHR levels are positively associated with both the relative and absolute risk of CMD, and this association is influenced by disease category, gender, age, and the presence of hypertension. Moreover, this association is independent of single-point NHHR levels.

Previous research has shown that elevated NHHR levels are a risk factor for CMD. Both cross-sectional and cohort studies have found that high NHHR levels are associated with the risk of developing new-onset diabetes (11) and CVD (12, 18). Unlike previous studies, we treated CMD as a single endpoint and, for the first time, demonstrated the impact of high NHHR on CMD, with a dose-response relationship. Each 1-SD increase in cum NHHR was associated with a 10% increase in the risk of new-onset CMD, independent of single-point NHHR levels. Not only did the relative risk increase, but compared to the Q1 group, the 5-year and 10-year absolute risk of CMD in the Q4 group exceeded 3% and 8%, respectively. Our findings have significant public health implications: reducing NHHR levels could not only lower the relative risk of CMD but also reduce absolute risk and the overall disease burden associated with CMD.

We also found that the association between high cum NHHR levels and CMD risk varies depending on disease category. The risk of ischemic heart disease was significantly higher than that of ischemic stroke. Each 1-SD increase in cum NHHR was associated with a 29% increase in the risk of myocardial infarction and revascularization, but only an 8% increase in the risk of ischemic stroke. Meta-analyses have also shown that the association between high NHHR levels and coronary heart disease is stronger than that with ischemic stroke (14, 19). Additionally, our study found an approximate U-shaped association between cumulative NHHR exposure and the risk of cerebral hemorrhage, a finding consistent with the U-shaped relationship between LDL-C and cerebral hemorrhage observed by Ma Chaoran et al. (20).

This study found that the association between cum NHHR exposure levels and the absolute risk of diabetes was stronger than that for CVD. In the Q4 group, the 5-year and 10-year absolute risks of developing diabetes were 7.01% and 13.42%, respectively, whereas the 5-year and 10-year absolute risks of developing CVD were only 3.97% and 8.62%. Previous studies have shown that high NHHR levels increase the risk of both diabetes (21) and CVD (12, 22). However, these results came from different studies with variations in study populations and designs, which could result in competitive effects. Thus, it is difficult to directly compare the risks of CVD and diabetes caused by NHHR. In our study, we used the same cohort and design to calculate the absolute risks of cumulative NHHR for CVD and diabetes, and we found that the absolute risk of diabetes was higher.

Additionally, we observed that the association between high cum NHHR exposure levels and CMD risk was more pronounced in low-risk populations such as women, individuals under 45 years old, and those without hypertension. For every 1 SD increase in cum NHHR, the risk of CMD increased by 13% in women, compared to 9% in men. This is consistent with findings from the ATTICA study (12), which showed that elevated NHHR levels increased the 10-year risk of cardiovascular disease more in women than in men. Previous studies have also indicated that elevated NHHR levels increase the risk of diabetes more in individuals under 55 years old compared to those aged 55 years or older (21), which aligns with our results. We found that CMD risk was higher in individuals under 45 years old compared to those aged 45 and older. Among individuals without hypertension, the risk of developing CMD increased by 13%, compared to an 8% increase in those with hypertension. This could be due to other competing risk factors in older individuals and those with hypertension, which may mask the impact of NHHR on CMD risk. Therefore, our findings suggest that in populations with high cum NHHR levels, prevention efforts for CMD should focus particularly on women, individuals younger than 45, and those without hypertension.

The mechanisms underlying the impact of cum NHHR exposure on CMD remain unclear, but there are several potential explanations. On one hand, NHHR has been shown to be significantly associated with insulin resistance (23) and atherosclerosis (24–26). Promoting inflammatory states, disrupting vascular smooth muscle and macrophage function, damaging endothelial cells, and accelerating the onset and progression of atherosclerosis (27–29). On the other hand, NHHR has been negatively correlated with the formation of coronary collateral circulation. Potentially increasing the risk of cardiovascular diseases (30–33), and reducing long-term survival. Furthermore, NHHR reflects cholesterol levels in both atherosclerotic and anti-atherosclerotic lipoproteins (22), representing the balance of cholesterol transport. Increased cholesterol transport to peripheral cells may lead to pancreatic cell dysfunction, causing hyperglycemia, which interferes with various metabolic pathways. This promotes lipid glycation, the formation of advanced glycation end products, and activation of the protein kinase C (PKC) pathway (34). Damaging blood vessels and peripheral nerves (26), thereby increasing the risk of cardiometabolic diseases (10, 24).

This study has several strengths. First, Unlike previous studies that mostly measured NHHR at a single time point, this study used cumulative NHHR exposure based on three measurements, reflecting long-term average exposure levels and providing more reliable results. Second, the study is based on the Kailuan cohort, which has a large sample size, high follow-up quality, and robust data. However, there are some limitations: First, The study population mainly consists of occupational workers, this may not be representative of all populations, and selection bias may exist. The study results need further validation in other populations. Moreover, the proportion of males in the study population is high, which may also limit the generalizability of the findings. Second, while we adjusted for various lifestyle factors and medical histories, some potential confounders, such as environmental changes, were not controlled for. Therefore, further large-scale prospective studies are needed to confirm these findings.

In conclusion, this study found that high cum NHHR exposure is a risk factor for CMD, independent of single-point NHHR levels. These findings provide new epidemiological evidence for the prevention of CMD. Therefore, in CMD prevention, attention should not be limited to traditional lipid markers, as cum NHHR also plays an important role in guiding preventive strategies.

## Data Availability

The original contributions presented in the study are included in the article/Supplementary Material, further inquiries can be directed to the corresponding author.
