# Supplementary material for: Association of cumulative non-high-density lipoprotein cholesterol to high-density lipoprotein cholesterol ratio with the risk of cardiometabolic disease
Source: Front Cardiovasc Med. 2024 Nov 20;11:1500025. doi: 10.3389/fcvm.2024.1500025 (PMC11614763; doi:10.3389/fcvm.2024.1500025)
Supplement: Supplementary file 1 [file Table1.pdf]

Supplementary Table 1. Problems 10th Revision (ICD-10) coding of the disease

Diseases	ICD-10
Myocardial infarction	I21
Ischemic stroke	I63
Cerebral hemorrhage	I61
Subarachnoid hemorrhage	I60
Revascularization	Z95
Type 2 diabetes	E11
